# Accurate Phylogenetic Tree Reconstruction from Quartets: A Heuristic Approach

**DOI:** 10.1371/journal.pone.0104008

**Published:** 2014-08-12

**Authors:** Rezwana Reaz, Md. Shamsuzzoha Bayzid, M. Sohel Rahman

**Affiliations:** Department of Computer Science and Engineering, BUET, Dhaka, Bangladesh; Pennsylvania State University, United States of America

## Abstract

Supertree methods construct trees on a set of taxa (species) combining many smaller trees on the overlapping subsets of the entire set of taxa. A ‘quartet’ is an unrooted tree over 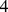 taxa, hence the quartet-based supertree methods combine many 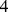-taxon unrooted trees into a single and coherent tree over the complete set of taxa. Quartet-based phylogeny reconstruction methods have been receiving considerable attentions in the recent years. An accurate and efficient quartet-based method might be competitive with the current best phylogenetic tree reconstruction methods (such as maximum likelihood or Bayesian MCMC analyses), without being as computationally intensive. In this paper, we present a novel and highly accurate quartet-based phylogenetic tree reconstruction method. We performed an extensive experimental study to evaluate the accuracy and scalability of our approach on both simulated and biological datasets.

## Introduction

A phylogenetic tree of a group of species (taxa) describes the evolutionary relationship among the species. The study of phylogeny not only helps to identify the historical relationships among a group of organisms, but also supports some other biological research such as drug and vaccine design, protein structure prediction, multiple sequence alignment and so on [1]. The ultimate goal of this research community is to infer the *Tree of Life*, the phylogeny of all living organisms on earth, provided that it exists.

Phylogenetic tree reconstruction by analyzing the molecular sequences of different species can be regarded as the *sequence-based* reconstruction of the phylogeny. Sequence-based phylogenetic methods are basically of three types [1]: (a) distance-based methods, such as Neighbor Joining (NJ) [2], which has very fast practical performance; (b) heuristics for either Maximum-Likelihood (ML) [3] or Maximum-Parsimony (MP) [4], which are two NP hard optimization problems; and (c) the Bayesian Markov Chain Monte Carlo (MCMC) method, which, instead of a single tree, produces a probability distribution of the trees or aspects of the evolutionary history. Sequence-based methods are generally highly accurate. However, these methods are computationally intensive. As a result, these can only be applied on small to moderate sized datasets if we want to provide results having an acceptable level of accuracy within a moderate amount of time. For larger datasets (few hundreds of taxa (species)), these methods may need several weeks or months to provide results with an acceptable level of accuracy [1]. As the amount of molecular data is accumulating exponentially with the continuous advancement in sequencing technologies, scientists are facing new computational challenges to analyze these enormous amount of data. Therefore, we are forced to rely on *supertree* methods, where smaller trees on overlapping groups of species are combined together to get a single larger tree. Supertree-based tree construction is a two-phase method: in the first phase, many small trees on overlapping subsets of taxa are constructed using a sequence-based method; and in the next phase the small trees are summarized into a complete tree over the full set of taxa.

Supertree methods are considered to be the likely solutions towards assembling the *Tree of Life*. Hence, these methods have drawn potential research interest in recent years. Supertree methods have two major motivations: firstly, it gives us the opportunity to achieve increased scalability and secondly, it is more suitable to combine the phylogenetic analyses on different types of data (e.g., molecular, morphological and gene-order data) or species groups. The careful design of supertree methods may allow us to work on very large (several hundreds taxa) datasets more accurately and easily. The most widely used supertree method is called the Matrix Representation with Parsimony (MRP) [5], [6]. MRP encodes all the small trees into a matrix using the characters 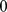, 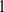 and 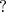. Then it uses Maximum-Parsimony (MP) [4] to get a tree from the data matrix. MRP is considered to be the most reliable supertree method to date. But since it uses an NP hard problem to analyze the data matrix, it is not efficient for large datasets.

*Quartet amalgamation* methods are supertree methods when each of the the small trees to be combined is a quatret, i.e., an unrooted tree having 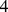 taxa. Quartet is the most basic piece of unrooted phylogenetic information. Quartet-based phylogenetic inference has drawn significant attention from the research community, and numerous quartet-based methods have been developed over the last two decades. In this paper, we present a novel and highly accurate quartet amalgamation technique. We conduct an extensive experimental study that demonstrates the superiority of our algorithm over QMC [7]–[9], which is known as the best quartet amalgamation method to date.

With the increasing abundance of molecular data, constructing species trees from multilocus data has become the focus of attention. But combining data on multiple loci is not a trivial task due to the gene tree discordance [10]–[12]. The task is even more complicated with the striking recognition that the most probable rooted gene tree topology (under a coalescent model [12]–[18]) need not match the species tree topology [19], [20]. These are termed as *Anomalous gene trees* (AGTs). AGTs occur because not all tree topologies are equiprobable under the coalescent model [18], [21], [22]. In fact, rooted AGTs exist for any species tree with 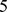 or more taxa. It has also been shown that rooted AGTs cannot occur with a three taxa and a symmetric four taxa species tree [19]. AGTs have also been studied for unrooted gene trees, and it has been observed that for a species tree with four taxa, the most probable rooted gene tree topologies have the same unrooted topology as the species tree [23]. This observation indicates that the most frequently occurring unrooted quartet is a consistent estimate of the unrooted species tree [23]. Thus, quartet based phylogeny can offer a sensible and statistically consistent approach to combine multilocus data, despite gene tree incongruence and AGTs [24], . Thus a highly accurate quartet amalgamation approach will help to design species tree estimation methods that are not susceptible to the gene tree discordance and AGTs. Notably, as has already been mentioned above, the other important advantage of quartet-based methods is that efficient design of such inference algorithm can be scalable to very large datasets (several hundreds or thousands of taxa).

### Previous Works

Quartet-based phylogenetic tree reconstruction has been receiving extensive attention in the literature for more than two decades. Different approaches have been proposed and improved time to time. Among these, the most prominent approaches are, quartet puzzling (QP), quartet joining (QJ) and quartet max-cut (QMC).

Quartet puzzling (QP) [26] infers the phylogeny of 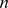 sequences using a weighting mechanism. First, it computes the maximum-likelihood values for the three topologies on every 4 taxa and uses these values to compute the corresponding probabilities. Using these probabilities as weights, the puzzling step constructs a collection of trees over 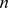 taxa. Finally it returns a consensus tree over *n*-taxa. TREE-PUZZLE [27] is a widely used program package that implements QP. In 1997, Strimmer et al. [28] extended the original QP algorithm by proposing three different weighting schemes, namely, continuous, binary and discrete. Later in 2001, Ranwez and Gascuel [29] proposed weight optimization (WO), an algorithm which is also based on weighted 4-trees inferred by using the maximum likelihood approach. WO uses the continuous weighting scheme defined in [28] and it searches for a tree on 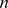 taxa such that the sum of the weights of the 4-trees induced by this tree is maximal [29]. Unlike QP, WO constructs a single tree over 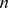 taxa; hence no consensus step is required. Though the speed and accuracy of WO are better than that of QP, its accuracy is lower than that of the methods based on evolutionary distances or maximum likelihood. Quartet joining (QJ) [30] was introduced in 2007 to overcome the limitations of QP and WO in outperforming the distance based methods. QJ provides the theoretical guarantee to generate the accurate tree if a complete set of consistent quartets is present. On average QJ outperforms QP and its performance is very close to the performance of NJ [2], but QJ outperforms NJ on quartet sets with low quartet consistency rate [30].

In 2008, Snir et al. [7] proposed a new quartet-based method, *short quartet puzzling* (SQP). The experimental studies in [7] shows that SQP provides more accurate trees than QP, NJ and MP. It differs from the previous techniques in that it does not require all three topologies of the quartets on every 4 taxa. It is able to construct the output tree from a subset of all possible quartets as input. This is a two-phase technique: the first phase uses the randomized technique for selecting input quartets from all possible 4-trees (estimated using ML), and the second phase uses Quartet Max Cut (QMC) [7], [8] technique for combining quartets into a single tree. The experimental study conducted by Swenson et al. [31] concludes that QMC performs better than the other supertree methods and MRP for smaller (100-taxon and 500-taxon) and high scaffold (i.e., high scaffold density) datasets. But MRP outperforms QMC and other supertree methods on larger and low scaffold (i.e., low scaffold density) datasets [31]. Subsequently, Snir and Rao presented a fast and scalable implementation of QMC [9], where they reported the improvement of QMC over MRP in terms of accuracy and running time. Although MRP is the mostly used supertree method in practice, the studies of [9], [31] suggest that QMC is so far the best quartet-based supertree method.

In this paper, we present a new quartet-based phylogeny reconstruction algorithm, *Quartet FM* (QFM), which uses a bipartition technique inspired from the famous Fiduccia and Mattheyses (FM) algorithm for bipartitioning a hyper graph minimizing the cut size [32]. As will be reported later, QFM is highly accurate and scalable to large datasets (upto several hundreds of taxa). We demonstrate the accuracy of QFM by analyzing its performance on both simulated and biological datasets. We have compared our method on simulated datasets with Quartet MaxCut (QMC) [7]–[9], and showed the superiority of our method over QMC in terms of the accuracy of the estimated trees. To show the potential of our method, we also analyzed a real biological dataset containing 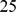 species from 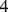 genera of birds (*Amytornis*, *Stipiturus*, *Malurus* and *Clytomias*). We have demonstrated a qualitative analysis of our results on real dataset based on the results of some rigorous previous studies on the same dataset.

### Problem Definition

We address the problem of *Maximum Quartet Consistency* (MQC), which is a natural optimization problem. This problem takes a quartet set 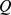 as the input and finds a phylogenetic tree 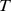 such that the *maximum* number of quartets in 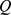 become “consistent” with 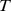 (or 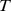 “satisfies” the maximum number of quartets). Now we formally define the problem.

### Problem 1 Maximum Quartet Consistency

***Input:*** *A multiset of quartets* 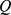 *on a taxa set* 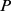.

***Output:*** *A phylogenetic tree* 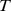 *on* 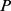 *such that* 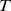 *satisfies the maximum number of quartets of* 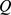.

The Maximum Quartet Consistency (MQC) problem is an NP-hard optimization problem [33]. Both exact and heuristic approaches are available for the MQC problem in the literature [34]. The running time of an exact algorithm grows exponentially with the increase of number of taxa, since the number of possible trees grows more than exponentially with the number of taxa [35]. So for larger datasets we have to resort to the heuristic solutions. The focus of this work is on heuristic solutions for the MQC problem as we aim to build the phylogenetic tree for several hundreds of taxa.

## Results

We have conducted an extensive experimental study on both simulated and biological datasets. We have evaluated the accuracy of the trees estimated by QFM and compared the results to that of QMC [9]. QMC is the most accurate quartet amalgamation method developed to date, and was shown to be more accurate than MRP [9]. We have reported RF (Robinson Foulds) [36] rates of the estimated trees. RF rate is the mostly used error metric, which is the ratio of the sum of the number of false positive and false negative edges to a factor 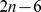, where 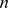 is the number of taxa [1]. The false positive (FP) and false negative (FN) edges are respectively, the edges which are absent in the true tree but present in the estimated tree, and the edges which are present in the true tree but absent in the estimated tree.

### Simulated Datasets

To investigate the performance of our method on various model conditions, we have generated quartet sets, taken uniformly at random from model trees, by varying the number of taxa (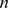), the number of quartets (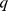) and the percentage of consistent quartets (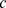) with respect to the model tree (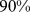 consistency level means that 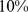 quartets are flipped to disagree with the model tree). We have generated model species trees with 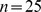, 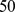, 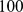, 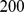, 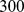, 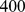 and 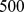 taxa. To generate the model trees and the input quartet sets, we have used the tool developed and used in [9]. The tool takes as input the number of taxa (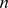), number of quartets (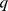) and the consistency level (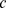), and returns the quartet sets accordingly. For 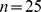, 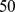, 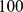, we have generated 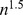, 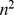 and 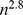 quartets. We have not generated more quartets because 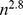 quartets have been empirically shown to be enough to construct very accurate phylogenetic trees [9]. Although 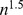 is a small number, we have chosen this size to test the performance of both methods on a comparatively smaller number of quartets as well. For 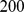, 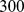, 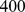 and 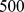-taxon model trees, we have generated datasets with 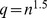 and 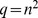. For each size (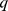), we have varied the percentage of consistent quartets (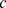) by making it 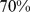, 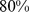, 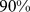, 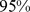 and 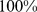. Thus in total we have generated 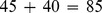 model conditions. To test the statistical robustness, we have generated 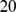 replicates of data for each of these model conditions. For each model condition, we report the average RF rate over the 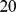 replicates of data. We also report the standard error, given by 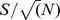 where 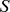 is the standard deviation and 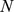 is the number of datapoints (which is 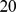 in our experiments). The standard errors are reported in Table S1 and Table S2 in File S1. We have used Wilcoxon signed-rank test with 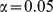 to test the statistical significance of the differences between QFM and QMC. The results of the Wilcoxon T-test (*p*-values) are reported in Table S3 in File S1.

### Analyses on the Simulated Datasets

**Figure 1:**
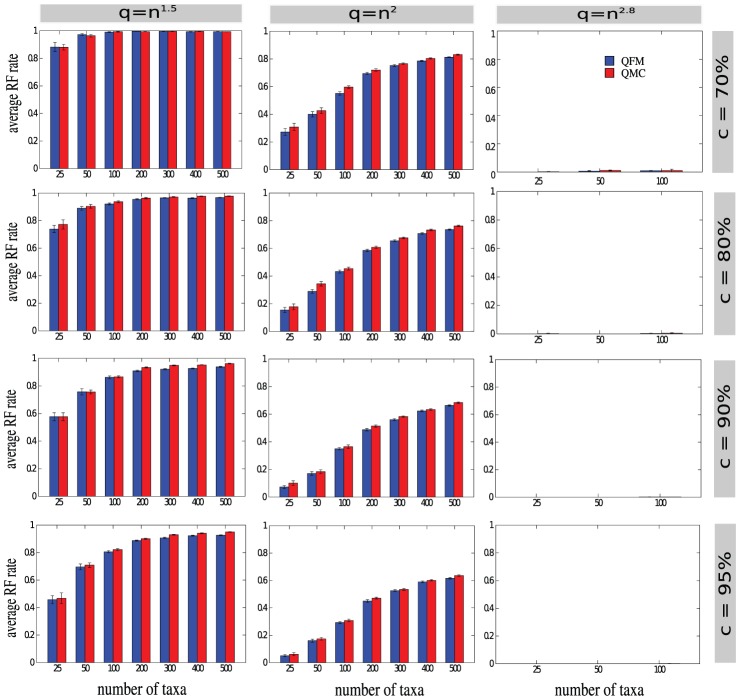
Average RF rates of QFM and QMC on the simulated datasets. We show average RF rates (over 20 replicates of data) for each model condition. We varied the number of taxa (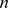), number of quartets (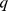) and the percentage of consistency level (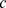). For a particular value of 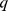 and 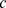, the number of taxa is varied along the X-axis, the average RF rate is shown along the Y-axis, and the error bars represent the standard errors. From left to right: the number of quartets are 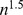, 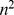, and 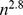. From top to bottom: 70%, 80%, 90% and 95% of the input quartets are consistent with the model species tree. We did not run our method on 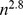 quartets when the number of taxa is more than 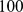, since these are computationally intensive and QFM could not be run within a reasonable time limit. Moreover, these model conditions are less revealing and interesting since both QMC and QFM can reconstruct the true species trees with 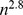 quartets.

We now present the results on the simulated datasets mentioned above. In each case, we have compared the average RF rate for the trees estimated by QFM and QMC. The results for 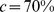, 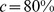, 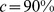 and 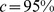 are summarized in Table 1. Figure 1 shows the bar charts comparing the values presented in Table 1. The results in Table 1 is presented in batches for different values of 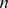 as follows. For 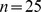, 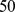, 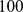, we have three rows, one each for 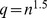, 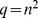 and 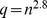. For 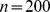, 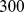, 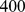, 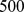 we have two rows, one each for 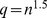 and 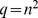. The topmost row of each batch of Table 1 shows the results when 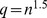 (from left to right, the consistency levels reported are 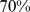, 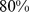, 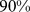, 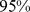, respectively). For this (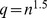) case, both QMC and QFM have performed poorly which implies that 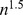 quartets are quite insufficient for accurate phylogeny reconstruction. This can be attributed to the fact that 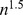 is a very small number compared to 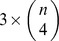 (i.e., the possible number of quartets). However, as the consistency level (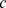) increases, QFM starts to produce better trees than QMC; and very often the improvements of QFM over QMC are statistically significant (see Table S3 in File S1). This is very promising in the sense that, QFM can construct more accurate trees than QMC even with very small number of quartets. The second row of each batch of Table 1 shows the results with 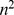 quartets. With 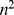 quartets, both QFM and QMC begin to produce better trees than that of 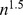 quartets. However, quadratic number of quartets is still not sufficient for reconstructing an accurate tree (which confirms the observation of [9]). But as before, QFM is statistically significantly better than QMC in most of the cases. The bottom most row of the first three batches in Table 1 shows the results with 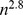 quartets. In this case, both QFM and QMC reconstruct highly accurate species trees (error rates are close to zero) even with 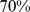 consistent quartets.

**Table 1:** Comparison of QFM and QMC under various model conditions.

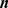	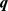	Average RF rate							
		c = 70%		c = 80%		c = 90%		c = 95%	
		QFM	QMC	QFM	QMC	QFM	QMC	QFM	QMC
25	125	0.882	0.881	**0.739**	**0.772**	0.577	0.577	**0.458**	**0.468**
25	625	**0.272**	**0.308**	**0.155**	**0.178**	**0.073**	**0.101**	**0.051**	**0.062**
25	8208	0	**0.002**	0	**0.002**	0	0	0	0
50	354	0.973	0.964	**0.890**	**0.904**	0.757	0.756	**0.696**	**0.709**
50	2500	**0.400**	**0.426**	**0.289**	**0.344**	**0.171**	**0.184**	**0.161**	**0.174**
50	57164	**0.007**	**0.011**	0	0	0	0	0	0
100	1000	**0.991**	**0.993**	**0.921**	**0.937**	**0.862**	**0.866**	**0.806**	**0.822**
100	10000	**0.551**	**0.597**	**0.433**	**0.454**	**0.350**	**0.365**	**0.293**	**0.308**
100	398108	**0.009**	**0.010**	**0.003**	**0.004**	0.001	0.001	**0**	**0.001**
200	2829	0.997	0.994	**0.955**	**0.963**	**0.909**	**0.934**	**0.887**	**0.901**
200	40000	**0.695**	**0.720**	**0.585**	**0.608**	**0.488**	**0.514**	**0.450**	**0.471**
300	5197	0.996	0.996	**0.965**	**0.972**	**0.921**	**0.949**	**0.907**	**0.930**
300	90000	**0.752**	**0.766**	**0.655**	**0.676**	**0.561**	**0.583**	**0.526**	**0.535**
400	8000	**0.993**	**0.996**	**0.963**	**0.977**	**0.926**	**0.952**	**0.923**	**0.941**
400	160000	**0.786**	**0.804**	**0.707**	**0.731**	**0.624**	**0.634**	**0.590**	**0.601**
500	11181	0.994	0.993	**0.967**	**0.978**	**0.938**	**0.962**	**0.926**	**0.950**
500	250000	**0.813**	**0.832**	**0.736**	**0.762**	**0.663**	**0.684**	**0.616**	**0.636**

Average RF rates of QFM and QMC over the 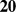 replicates of data under various model conditions. We varied the number of taxa (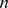), the number of quartets (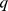), and the percentage of consistent quartets (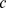). Results are shown in bold face where QFM is better than QMC.

From these results, it is clear that QFM either matches the accuracy of QMC or (in most cases) produces better trees than QMC. QFM outperforms QMC in 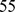 cases out of the 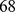 model conditions shown in Table 1, and in 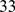 cases the differences are statistically significant (see Table S3 in File S1). QMC is better than QFM on only 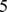 cases, but the differences between the two methods are not statistically significant. For the rest 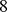 cases, both QFM and QMC have equal error rates (these are mostly the datasets with 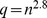 quartets where both of them have been able to reconstruct the true trees).

We have also evaluated QFM and QMC on the noise-free model conditions, meaning that all the quartets are accurate (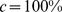). Table 2 demonstrates the results under the parameters (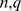) with 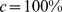. Of the 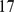 model conditions analyzed, QFM has been found to be better than QMC on 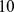 cases, and the improvements are statistically significant in 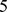 cases (see Table S3 in File S1). QMC is better than QFM in two cases but the differences are not statistically significant. In 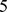 cases QFM and QMC have identical accuracy.

**Table 2:** Comparison of QFM and QMC under the noise-free model conditions.

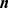	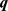	Average RF rate	
		c = 100%	
		QFM	QMC
25	125	**0.444**	**0.515**
25	625	0.056	0.052
25	8208	0	0
50	354	**0.661**	**0.666**
50	2500	0.140	0.140
50	57164	0	0
100	1000	**0.777**	**0.797**
100	10000	**0.269**	**0.274**
100	398108	0	0
200	2829	**0.848**	**0.881**
200	40000	0.424	0.424
300	5197	**0.887**	**0.907**
300	90000	0.506	0.499
400	8000	**0.897**	**0.930**
400	160000	**0.554**	**0.555**
500	11181	**0.903**	**0.937**
500	250000	**0.590**	**0.606**

Average RF rates of QFM and QMC over the 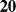 replicates of data under the noise-free model conditions (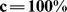). We varied the number of taxa (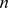) and the number of quartets (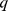). Results are shown in bold face where QFM is better than QMC.

### Computational Issues

We have evaluated the running time and memory usage of QFM and QMC. On smaller datasets, both QFM and QMC run in few seconds. For example, on 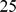 taxa, QFM took between 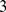 seconds to 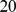 seconds (depending on the number of quartets), and QMC took less than 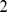 seconds. Both of these methods are very fast on the datasets with up to 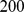 taxa and with 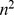 quartets: QFM took few minutes while QMC completed in few seconds. However, QFM is much slower than QMC on the larger datasets. For example, QFM took 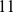 hours for the largest datasets of our experiment with 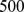 taxa and 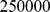 quartets, while QMC took only one minute. We believe that this difference is due to the naive implementation of our algorithm. QMC has been implemented in a very efficient code, and it scales well on larger datasets. We are currently working on improving our implementation using advanced data structures. We are also parallelizing our divide and conquer based approach.

We have also measured the memory usage by these methods. Both QFM and QMC are memory efficient and use only few megabytes of memory. For example, the peak memory usages by QMC and QFM on the datasets with 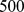 taxa and 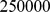 quartets are 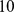 MB and 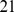 MB, respectively.

### Analyses on the Avian Biological Dataset (Australo-Papuan Fairy-wrens)

We have further evaluated the performance of QFM on a real avian biological dataset consisting of 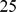 birds. Since Avian phylogeny is considered to be hard to reconstruct, we have chosen this dataset as a good representative of real datasets. This dataset consists of 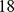 gene trees on 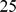 species representing 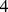 genera of birds (*Amytornis*, *Stipiturus*, *Malurus* and *Clytomias*) from Australo-Papuan avian family Maluridae, obtained from TreeBASE [37]. This dataset has originally been used to study the efficacy of species tree methods at the family level in birds, using the Australo-Papuan Fairy-wrens (Passeriformes: Maluridae) clade [38]. Due to the presence of substantial amount of incomplete lineage sorting (ILS) [38], analyzing this family of birds is quite challenging.

**Figure 2:**
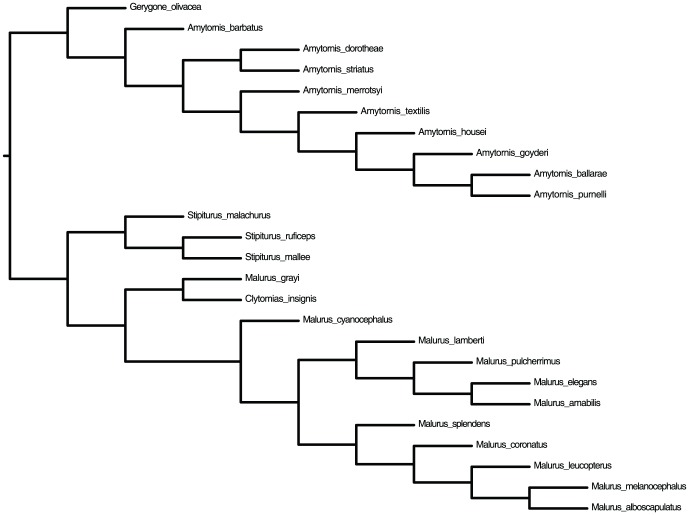
The 25 species avian phylogeny, representing 4 genera of birds from Maluridae family, estimated by QFM using the 227,700 embedded quartets in 18 gene trees. The evolutionary relationships maintained by this tree are supported by the findings of the previous studies [38], [40], [41], [43].

We have decomposed every gene tree into its induced quartets which is called *embedded quartets* [9], [39]. Then, we have taken the union of all these quartets (multiple copies of a quartet have been retained). In this way we get 227,700 quartets. We have used these quartets to estimate a species tree using our method (QFM). We also ran QMC on this datasets. Both QFM and QMC returned the same tree. The tree is shown in Figure 2.

Since we do not know the true trees for biological datasets, we have compared the result obtained from QFM with biological beliefs and other rigorous analyses. The tree returned by QFM (which is identical to the tree estimated by QMC) is quite interesting and consistent with the previous findings as discussed below.

• QFM has been able to correctly identify the clusters associated with the four genera of birds. Also, it has placed the group of *Amytornis* birds as the sister to the rest of the family, and the group of *Stipiturus* birds as the sister to *Malurus* and *Clytomias* birds. These evolutionary relationships maintained by QFM are supported by the findings of the previous studies [38], [40], [41].

• *Amytornis*: Using allozyme analysis, Christidis [41] has shown that *A. barbatus* is the earliest diverged lineage in the Amytornis genus. Same results have been obtained by a DNA sequencing study in [42]. The sequence-based analysis of Lee et al. [38] also have confirmed this. Our analyses with QFM also have found the same pattern. Lee et al. [38] also have shown that *A. housei* should be within the *textilis* complex, which is confirmed by our QFM tree.

• *Stipiturus*: Evolutionary relationships within the *Stipiturus* genus have been well studied [38], [40], [43]. Our study is consistent with the previous findings: *S. mallee* and *S. ruficeps* are closer to each other than they are to *S. malachurus*.

• *Clytomyias* and *Malurus*: *C. insignis* was placed to *Stipiturus* species by [40]. However, in a more recent extensive multi-locus study, Lee et al. [38] argued that *C. insignis* is closer to *M. grayi*. Our study has also confirmed this fact. Also our study has confirmed their [38] findings that *M. alboscapulatus* is closer to *M. melanocephalus* than to *M. leucopterus*.

Lee *et al.* [38] showed that ILS is likely a general feature of the genetic history of these avian species. Since quartets are not prone to anomaly zone [19], [23], quartet based analyses to resolve the avian history is of high importance. Interestingly, both QMC and QFM resolved the evolutionary history of these 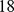 birds similarly. Therefore, we believe that this tree should be considered as a reasonable hypothesis about the evolutionary history of this family of birds.

## Discussion

In this work we have presented a novel and highly accurate quartet amalgamation technique, which we refer to as QFM. We have demonstrated the superiority of our method over QMC, which is known to be the best quartet amalgamation method to date.

QFM is a new promising divide and conquer supertree method having an algorithmic appeal. We have conducted an extensive experimental study comparing QFM against QMC under different model conditions by varying different parameters. For almost all model conditions considered, QFM performs at least equal but in most cases better than QMC. In line with the experimental results shown in [9], we have found that quadratic sampling of quartets is not sufficient for accurate supertree construction. However, with 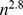 quartets, both QFM and QMC can reconstruct very accurate trees indicating that it is possible to reconstruct an accurate supertree from large number of quartets, even with high amount of noise in the input data. QFM has also been tested on real biological datasets and has been shown to perform pretty well. The tree estimated by QFM has maintained the important evolutionary relationships despite the presence of incomplete lineage sorting. This is particularly interesting because this suggests that we can use quartet-based technique to develop species tree estimation method (from multi-locus data), which is less susceptible to gene tree incongruence due to ILS.

Species tree estimation is frequently based on phylogenomic approaches that use multiple genes from throughout the genome. However, combining data on multiple genes is not a trivial task. Genes evolve through biological processes that include deep coalescence (also known as incomplete lineage sorting (ILS)), duplication and loss, horizontal gene transfer etc. As a result the individual gene histories can differ from each other [10]. Species tree estimation in the presence of ILS is a challenging task. Moreover, anomalous gene trees (AGTs) make this task even more complicated [19], [20]. It has been proven that AGTs cannot occur in quartets and thus the most probable quartets induced by the true gene trees represent the true species trees for the corresponding four species [19], [23], Therefore, quartets can be used to design statistically consistent methods (methods that have the statistical guarantee to construct the true species tree given sufficiently large number of true gene trees) for constructing the species tree from gene trees (which evolve with ILS) as follows. First, we compute the quartets induced by the gene trees. For every four species, there are three possible quartets. Given sufficiently large number of true gene trees, the most probable quartets (the most frequently occurring quartets) on every four species represent the true species trees for those four species. Thus combining the most probable quartets to get a single and coherent species tree is an statistically consistent approach for species tree estimation. In this context, we can formalize the maximum weighted quartet satisfiability problem as follows.

• ***Input:*** A set 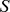 of weighted quartets.

• ***Output:*** The species tree 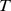 such that 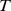 maximizes the summation of the weights of the satisfied quartets in 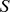.

We can define the weight of a quartet 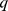 as the proportion of the gene trees that induce 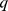. We can also incorporate the branch lengths in defining the weights. One major advantage of QFM is that it can readily be adapted to take a set of weighted quartets as input without making any change in its algorithmic constructs. Therefore, we think QFM is an important contribution to the phylogenomic analyses, in particular for estimating species trees from a set of gene trees where gene trees can be discordant from each other due to ILS.

Another advantage of QFM lies in its flexibility in choosing the *partition score* function (see “Partition Score” section). QFM can be customized to take different scoring functions (i.e., 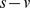, 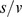, etc.) without making any change in the algorithmic construct. We have observed that QFM may not give the same result for different scoring functions for the same dataset. So for different datasets, we may obtain better results by adapting different suitable scoring functions. Thus QFM provides us with the flexibility to change the scoring function as needed. In future we shall try to make our algorithm self-adaptable to the appropriate scoring function by analyzing different characteristics of the input datasets. Notably, as has already been discussed above, one shortcoming of the current implementation of QFM is that it is not as fast as QMC.

## Materials and Methods

In this section we present our heuristic algorithm, namely, the **Q**uartet **FM** (**QFM**) algorithm. Our algorithm employs a *quartet based supertree reconstruction* technique that involves a bipartition method inspired by the Fiduccia Mattheyses (FM) bipartition technique [32].

### Basics

**Figure 3:**
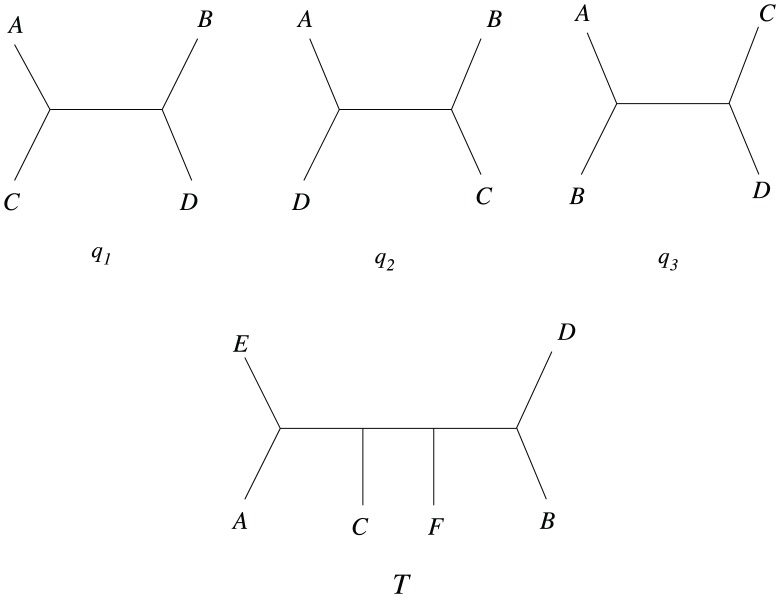
Quartet consistency with a tree 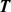. Among the three quartets, only 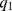  =  ((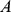, 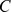), (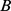,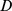)) is consistent with 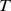 because 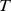 has an internal edge that separates taxa 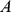 and 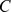 from taxa 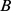 and 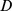 in 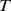.

A quartet 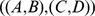 is *consistent* with a tree 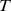 if in 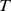, there is an edge (or path in general) separating 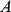 and 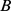 from 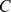 and 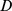. For any four taxa, only one quartet (out of 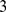 possible quartets) will be consistent with a tree 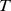. In Figure 3 among the three quartets, quartet 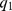 is consistent with tree 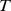 as there exists an edge in 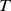 such that it separates 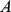 and 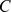 from 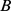 and 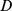. Other two quartets are inconsistent with 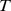 as no such edge exists in 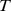.

A bipartition of an unrooted tree 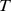 is formed by taking any edge in 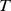, and writing down the two sets of taxa that would be formed by deleting that edge. Let 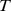 be a tree over the taxa set 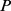. Now, if we take an internal edge 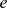 of 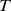 and delete 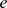, then we get two subtrees, namely, 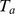 and 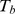. Let 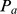 and 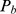 be the sets of taxa of 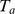 and 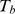 respectively. We shall denote such bipartition by (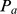, 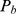). Thus an internal edge in 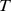 corresponds to a bipartition of 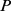.

A quartet 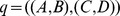 is *satisfied* with respect to a bipartition 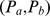 if taxa 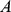 and 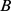 reside in one part and taxa 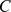 and 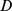 reside in the other. A *satisfied* quartet is *consistent* with 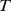. The quartet 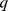 is said to be *violated* with respect to a bipartition 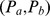 when taxa 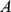 and 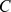 (or 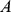 and 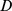) reside in one part and taxa 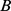 and 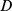 (or 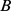 and 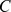) reside in the other part. On the other hand, 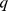 is said to be *deferred* with respect to a bipartition 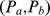 if any three of its four taxa reside in one part and the fourth one resides in the other.

A tree 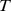 over a taxa set 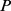 is said to be a *star*, if 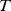 has only one internal node and there is an edge from the internal node incident to each taxon 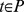. We shall refer to such a tree as a *depth one tree*.

### Divide and conquer approach

We follow a divide and conquer approach similar to QMC [7]–[9]. Let, 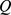 be a set of quartets over a set of taxa, 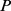. We aim to construct a tree 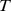 on 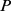, satisfying the largest number of input quartets possible. The divide and conquer approach recursively creates bipartition of the taxa set, where each bipartition corresponds to an internal edge in the tree under construction. QMC uses a heuristic bipartition technique which is based on finding a maximum cut (MaxCut) in a graph over the taxa set, where the edges represent the input quartets [9]. On the other hand, our algorithm uses a heuristic bipartition algorithm inspired by the famous Fiduccia and Mattheyses (FM) [32] bipartition algorithm.

#### Divide

At each recursive step, we partition the taxa set 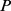 into two sets 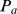 and 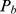. We shall describe the bipartitioning algorithm in “Method of Bipartition” section. After the algorithm partitions the taxa set, it augments both parts (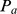 and 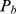) with a unique dummy (artificial) taxon. This taxon will play a role while returning from the recursion. After the addition of the dummy taxon to the sets 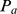 and 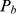, we subdivide the quartet set 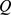 into two sets, 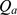 and 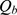. A quartet set 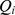 takes those quartets 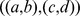 from 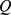 such that either all four taxa 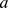, 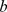, 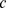 and 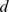 or any three thereof belong to 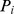 (here 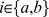). In other words, satisfied or violated quartets with respect to the partition 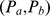 are not considered to be included in either 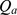 or 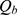. Moreover, in every deferred quartet, where three taxa are in the same part, the other taxon is renamed by the name of the dummy taxon, and the quartet continues to the next step. Thus we get, two 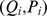 pairs: 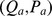 and 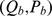. We then recurse on both pairs 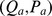 and 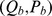 if 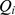 is non-empty and 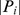 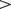 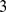. If either 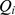 is empty or 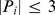, we return a *depth one tree* over the taxa set 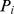.

#### Conquer

On returning from the recursion, at each step, we have two trees, 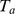 (corresponding to 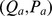) and 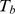 (corresponding to 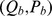). These two trees are rerooted at the dummy taxon. Then the dummy taxon is removed from each tree and the two roots are joined by an internal edge.

**Figure 4:**
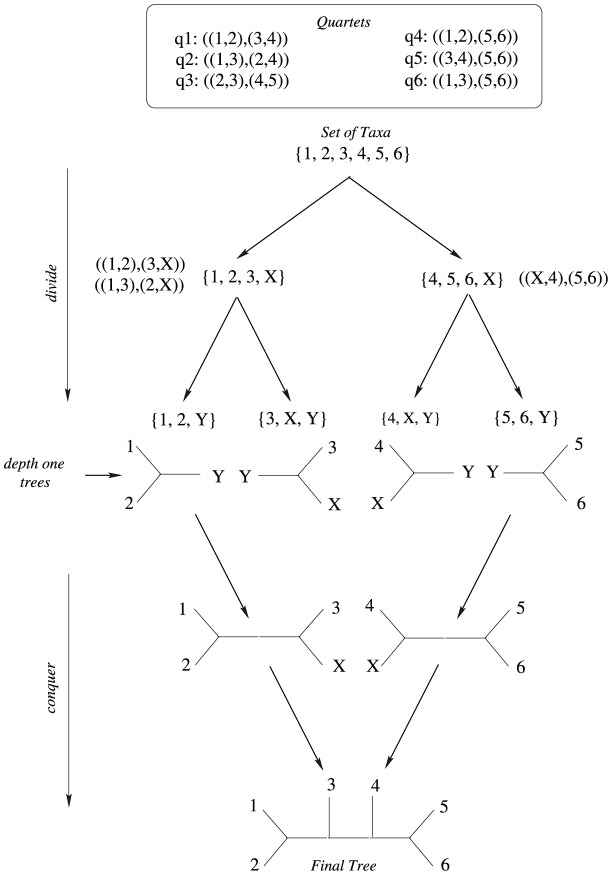
Divide and conquer approach. Divide: At each step, the input set of taxa of this step is partitioned into two sets and an unique dummy taxon is added to both sets. The input quartet set is then partitioned into two sets according to the bipartition of the set of taxa. So we get two (taxa set, quartet set) pairs, which are input to the successive divide steps. If at any step, the quartet set gets empty or the size of the taxa set becomes less than or equal to 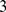, a depth one tree over the taxa set is returned. Conquer: At each step, there are two trees corresponding to the divide calls initiated at this step. These two trees are joined on the dummy taxon introduced at this step during divide. For example, the leftmost two depth one trees, when returned to its caller, are joined on the dummy taxon 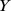.

Figure 4 describes the high level divide and conquer algorithm. Let 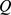 be the input quartet set and 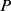 be the corresponding taxa set. Assume that 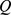  =  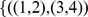, 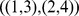, 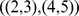, 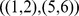, 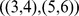, 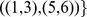, and hence 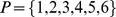. First, 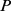 is partitioned into two sets, 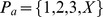 and 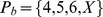 by using the bipartition technique described in “Method of Bipartition” section. Here, 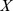 is the dummy taxon. The bipartition 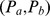 satisfies quartets 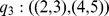, 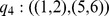 and 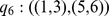 from 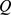. So these quartets will not be considered in the next level. 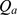 takes 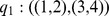 and 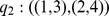 as three of the taxa of 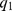 and 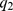 reside in 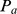. We replace the taxon which does not belong to 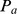 with the dummy taxon 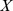. Hence we get 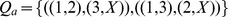. Similarly we get 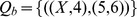. Next we recurse on 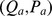 and 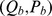, and 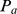 and 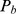 are partitioned further into 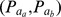 and 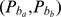, respectively. The partition 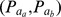 satisfies 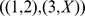 and violates 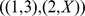 in 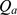 and 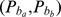 satisfies the only quartet in 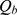. So the quartet sets for the next level are empty and hence no more recursion is required. We return a *depth one tree* for each of the taxa sets 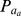, 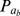, 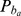 and 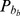. The returned trees are merged by removing the dummy taxon of that level and joining the branches of the dummy taxa. In Figure 4, the upper half shows the *divide* steps. The *depth one trees* are returned when no more recursion is required. The lower half of Figure 4 shows how the trees are returned and merged as the recursion unfolds (conquer step). Thus we get the final merged tree 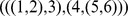 (shown at the bottom of Figure 4) satisfying 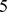 quartets in total. The satisfied quartets are 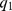, 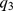, 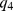, 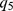 and 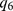.

### Method of Bipartition

The most crucial part of our algorithm is the bipartition (divide step) technique. Here, we differ from QMC [7]–[9] and adopt a new bipartition technique inspired by the famous Fiduccia and Mattheyses (FM) algorithm for bipartitioning a hyper graph minimizing the cut size [32]. In divide and conquer based phylogenetic tree construction, the bipartition of the taxa set corresponds to an internal edge of the tree under construction. An internal edge, in turn, plays a role to make quartets to be satisfied or violated against the bipartition. So we adopt a different bipartition technique from that used in QMC, with an objective to get better results.

Our bipartition algorithm takes a pair of taxa set and a quartet set (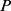, 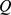) as input. It partitions 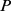 into two sets, namely, 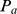 and 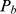 with an objective that (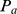, 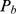) satisfies the maximum number of quartets from 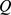. The algorithm starts with an initial partition and iteratively searches for a better partition. We will use a heuristic search to find the best partition. Before we describe the steps of the algorithm, we describe the algorithmic components.

#### Partition Score

We assess the quality of a partition by assigning a *partition score*. We use a scoring function, 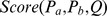, such that the higher score will indicate a better partition. This function checks each 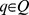 against the partition 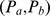 and determines whether 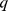 is *satisfied*, *violated* or *deferred*. We define the score function in terms of the number of satisfied and violated quartets. Let 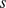 and 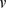 denote the number of satisfied and violated quartets. Then, two natural ways of defining the score function are: 1) taking the difference between the number of satisfied and violated quartets (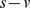), and 2) taking the ratio of the number of satisfied and violated quartets (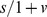). As num In this paper, we used 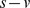 as the score function. We can also use some other complicated score functions defined in terms of the number of satisfied, violated and deferred quartets (i.e., 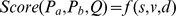, where 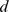 denotes the number of deferred quartets). In our preliminary experimental study, we have explored different score functions and observed that 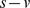 gives better performance in most of the cases. Notably, although in some cases other functions (e.g., 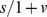, 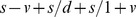) achieve better results than 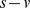 (results are not shown in this paper), none of them is consistently better than 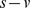.

#### Gain Measure

Let 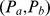 be a partition of set of taxa 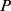. Let 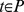 be a taxon and without loss of generality we assume that 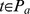. Let 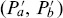 be the partition after moving the taxa 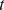 from 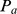 to 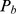. That means, 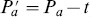, and 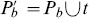. Then we define the *gain* of the transfer of the taxon 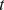 with respect to 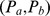, denoted by *Gain* 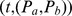, as 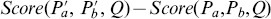.

#### Singleton Bipartition

A bipartition (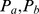) of 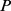 is *singleton* if 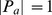 or 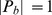. In our bipartition algorithm, we keep a check for the singleton bipartition. We do not allow our bipartition algorithm to return a singleton bipartition to avoid the risk of an infinite loop.

#### Algorithm

Now we describe the bipartition algorithm which we call MFM (Modified FM) Bipartition Algorithm. Let, (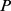, 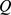) be the input to the bipartition algorithm, where 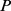 be a set of taxa and 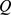 be a set of quartets over the taxa set 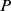. We start with an initial bipartition 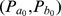 of 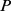. The initial bipartitioning is done in four steps.

• Step 1: We count the frequency of each distinct quartet in 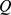.

• Step 2: We then sort 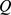 by the frequency count of the quartets in a decreasing order.

• Step 3: Suppose after sorting 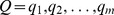, where 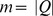. Now we consider the quartets one by one in the sorted order. Initially both 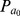 and 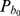 are empty.

Let 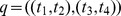 be a quartet in 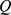. If none of the 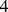 taxa belongs to either 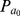 or 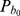, then we insert 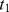 and 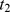 in 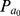 and 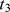 and 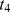 in 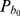. Otherwise, if any of the 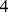 taxa exists in either 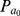 or 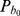 we take the following actions to insert a taxon which doest not exist in 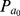 or 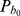. We maintain an insertion order. We consider 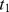, 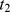, 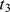 and 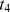 respectively.

– To insert 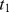, we look for the partition of 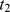 (if 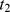 exists in any part) and insert 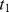 into that partition. But if 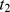 does not exist in either of the partitions, then we look for the partition of either 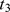 or 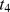 (either of these two must exist in 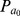 or 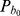) and insert 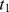 into the other partition.

– To insert 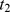, we look for the partition of 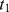 and insert 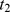 into that partition.

– To insert 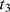, we look for the partition of 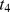 (if 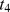 exists in any part) and inset 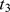 into that partition. But if 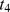 does not exist in either of the partitions, then we look for the partition of either 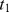 or 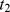 and insert 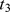 into the other partition.

– To insert 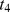, we look for the partition of 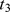 and insert 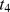 into that partition.

• Step 4: When we insert a taxon 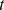 to any part, we remove it from 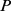. After considering each 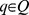 and inserting taxa accordingly, if 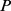 remains non-empty, we insert the remaining taxa to either part randomly.

**Figure 5:**
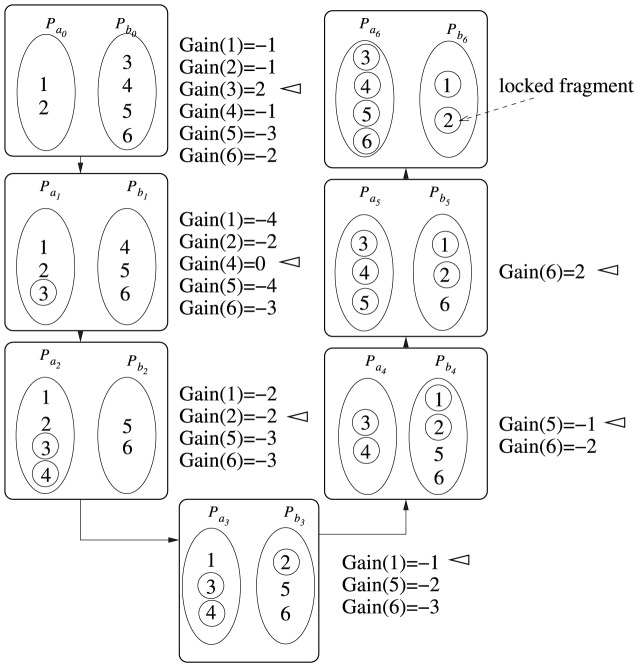
An example iteration of the Bipartition Algorithm MFM. The locked taxa are shown in circles. At each step, the taxon which has the maximum gain and will be transferred from its current partition to the other is indicated by a left arrow. (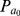, 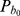) is the initial bipartition of this iteration. Initially all taxa are free (i.e, not locked). The gain is computed for each free taxon of this step and the taxon (which is 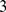 here) with maximum gain is transferred from its own partition to the other partition. Thus we get partition (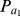, 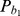), where 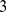 is a locked taxon. In this way, only one taxon is locked at a step and once a taxon is locked, it remains locked throughout the iteration. An iteration completes when all taxa get locked. Here, all taxa get locked at (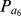, 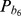).

Obtaining 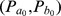, we search for a better partition iteratively. At each iteration, we perform a series of transfers of taxa from one partition set to the other to maximize the number of satisfied quartets. At the beginning of an iteration, we set the status of all the taxa as *free*. Then, for each *free* taxon 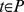, we calculate 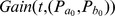, and find the taxon 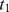 with the maximum gain. There can be more than one taxa with the maximum gain where we need to break the tie. We will discuss this issue later. Next we transfer 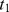 and set the status of this taxon as *locked* in the new partition that indicates that it will not be considered to be transferred again in this current iteration. This transfer creates the first intermediate bipartition 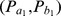. The algorithm then finds the next free taxon 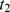 with the maximum gain with respect to 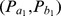, and transfer and lock that taxon to create another intermediate bipartition 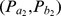. Thus we transfer all the free taxon one by one. Let 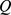 be the input quartet set and 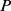 be the corresponding taxa set. Assume that 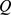  =  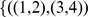, 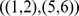, 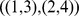, 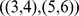, 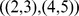, 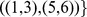 (same as used in Figure 4). Hence, 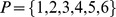. Following the steps of the initial bipartition, we get the initial bipartition 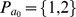 and 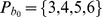. Figure 5 shows the first iteration of the bipartition algorithm for this particular example.

Suppose that the taxa are locked in the following order: 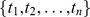. That is, 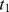 has been locked first, then 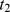, 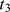 and so on. Let, the gain values of the corresponding partitions are:
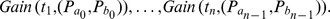



Now we define the cumulative gain up to the 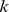
th transfer as
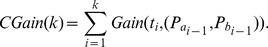



The maximum cumulative gain, 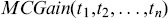 is defined as
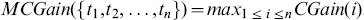



In each iteration, the algorithm finds the current ordering (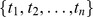) of the transfers and saves this order in a log table along with the cumulative gains (see Table 3 for example). Let 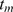 be the taxon in the log table corresponding to 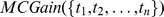. This means that we obtain the maximum cumulative gain after moving the 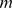
th taxon (with respect to the order stored in the log table). Then we rollback the transfers of the taxa (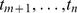) that were moved after 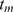. Let the resultant partition after these rollbacks is 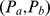. This partition will be the initial partition for the next iteration. In this way, the algorithm continues as long as the maximum cumulative gain is greater than zero and returns the resultant bipartition. Table 3 lists the order of locking, corresponding gain and cumulative gain with respect to the iteration illustrated in Figure 5. From Table 3 we note that we get the maximum cumulative gain, 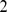, after moving taxon 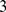. Here, we also get the maximum value of cumulative gain after moving taxon 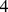. We break the tie arbitrarily. We consider the taxon for which we get the maximum cumulative gain for the first time. For this example, we get the maximum cumulative gain of 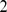 at taxon 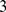 for the first time. So we rollback all the subsequent moves. The resultant partition after this rollback is 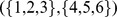 (partition 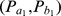 in Figure 5). Similarly, Table 4 lists the ordering of locking, corresponding gain and cumulative gain with respect to the iteration which follows the iteration illustrated in Figure 5. From Table 4 we get that the maximum cumulative gain is 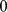. So the moves are rolled back and we get the final resultant partition 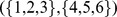.

**Table 3:** Gain Summary.

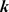	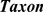	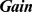	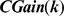
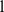	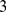	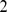	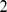
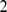	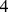	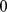	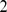
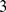	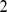	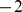	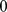
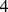	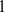	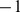	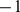
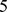	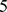	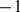	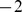
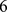	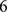	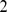	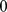

The log table corresponding to the iteration shown in Figure 5. Here 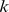 represents the step number. The input partition to step 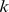 is (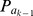, 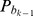). The second column shows the taxon that has the maximum gain at the corresponding step, and the third column shows the corresponding maximum gain. The fourth column shows the cumulative gain of the gains listed in the third column. We observe that the cumulative gain gets maximum (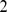) after moving taxon 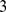 in step 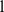. So all the subsequent moves of taxa are rolled back. The resultant partition of this iteration is (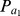, 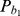)  =  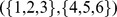, which is the initial partition for the next iteration of the iteration in Figure 5.

**Table 4:** Gain Summary.

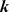	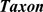	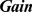	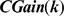
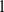	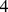	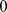	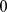
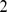	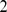	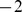	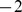
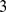	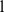	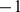	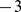
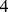	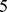	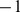	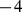
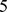	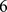	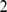	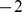
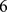	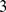	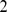	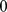

The log table corresponding to the next iteration of the iteration shown in Figure 5. Here 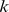 represents the step number. The input partition to step 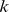 is (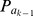, 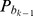). The second column shows the taxon that has the maximum gain at the corresponding step, and the third column shows the corresponding maximum gain. We observe that the cumulative gain gets maximum (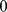) at step 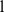. So we rollback all the subsequent moves including the move at step 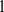 and return the initial partition 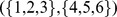 of this iteration as the resultant bipartition of the bipartition algorithm. No more iteration is needed as the maximum cumulative gain of the current iteration is not greater than zero.

As we have mentioned earlier, we do not allow any transfer of taxa that results into a singleton bipartition. Therefore, we need to add some additional conditions. Also, there could be more than one free taxa with the maximum gain, where we need to decide which one to transfer. We consider the following cases to address these issues. Let, 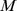 be a set of free taxa with the maximum gain.

• Case 1: 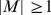 and at least one corresponding bipartition is not singleton. That means, there exists 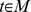 such that transfer of 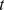 does not result into a singleton bipartition. Let 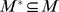 be the set of taxa, that can be safely transferred without resulting in a singleton bipartition. Note that, 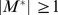. If 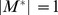, we transfer the taxa 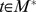. Otherwise, we have more than one taxa in 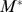. In that case, we pick the taxon 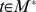, for which the corresponding bipartition (after transferring 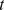) satisfies maximum number of quartets (note that every taxa in 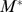 has the same gain, but the corresponding bipartitions do not necessarily satisfy the same number of quartets). In the case of a tie, we choose one taxon at random.

• Case 2: 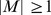 and transfer of each 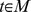 results in a singleton bipartition. In this case, we consider the set of taxa with the second highest maximum gain. Let 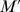 be the set of free taxa with the second highest maximum gain. We then recursively check ‘Case 1’ and ‘Case 2’ on 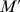. If we cannot find a taxon that can be transferred without resulting into a singleton bipartition, we make the status of all the free taxa *locked* and set their gain to zero.

At each divide step we have a 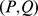 pair as input. The bipartition algorithm returns a bipartition 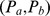 of the taxa set 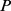. We then divide 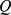 into 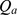 and 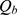 and obtain 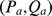 and 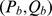 pairs. 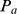 and 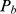 will be further bipartitioned in subsequent divide steps. The pseudo-code of the bipartition method MFM is given in Table S4 in File S1. Moreover, the run time analyses of Algorithm MFM is described in .

## Supporting Information

File S1 **Supplementary material.** Additional tables, and the pseudocode and time complexity of MFM bipartition algorithm are presented.
(PDF)
