# Supplementary material for: Accurate Phylogenetic Tree Reconstruction from Quartets: A Heuristic Approach
Source: PLoS One. 2014 Aug 12;9(8):e104008. doi: 10.1371/journal.pone.0104008 (PMC4130513; doi:10.1371/journal.pone.0104008)
Supplement: File S1 — Supplementary material. Additional tables, and the pseudocode and time complexity of MFM bipartition algorithm are presented. (PDF) [file pone.0104008.s001.pdf]

Supplementary Material for Accurate Phylogenetic Tree Reconstruction from Quartets: A Heuristic Approach

Rezwana Reaz*, Md. Shamsuzzoha Bayzid, M. Sohel Rahman
* E-mail: rimpi@cse.buet.ac.bd

Standard Error and Statistical Significance

Standard Error: For each model condition of the simulated dataset, we calculate the standard error of RF rates, given by $S/\sqrt(N)$, where S is the standard deviation and N is the number of datapoints (which is 20 in our experiments). Table S1 and Table S2 show the standard errors of RF rates of QFM and QMC over the 20 replicates of data under various model conditions.

Statistical Significance: We have used Wilcoxon signed-rank test with $\alpha = 0.05$ to test the statistical significance of the differences between the RF rates of QFM and QMC. The results are shown in Table S3.

Table S1. Standard error of QFM and QMC under various model conditions.

n	q	Standard error							
		c = 70%		c = 80%		c = 90%		c = 95%	
		QFM	QMC	QFM	QMC	QFM	QMC	QFM	QMC
25	125	0.032	0.020	0.026	0.034	0.029	0.029	0.029	0.039
25	625	0.025	0.026	0.019	0.021	0.011	0.017	0.008	0.012
25	8208	0	0.002	0	0.002	0	0	0	0
50	354	0.007	0.009	0.013	0.013	0.023	0.014	0.021	0.016
50	2500	0.019	0.021	0.014	0.016	0.013	0.014	0.011	0.009
50	57164	0.003	0.003	0	0	0	0	0	0
100	1000	0.003	0.003	0.007	0.007	0.010	0.007	0.008	0.007
100	10000	0.012	0.009	0.011	0.010	0.009	0.013	0.008	0.010
100	398108	0.002	0.010	0.001	0.004	0.001	0	0	0.001
200	2829	0.001	0.001	0.004	0.005	0.005	0.004	0.004	0.004
200	40000	0.009	0.009	0.008	0.009	0.009	0.008	0.010	0.007
300	5197	0.001	0.001	0.002	0.002	0.004	0.003	0.005	0.003
300	90000	0.007	0.006	0.007	0.006	0.007	0.005	0.007	0.007
400	8000	0.002	0.001	0.003	0.002	0.003	0.001	0.003	0.003
400	160000	0.004	0.004	0.006	0.006	0.006	0.006	0.006	0.005
500	11181	0.001	0.001	0.002	0.002	0.004	0.002	0.003	0.002
500	250000	0.003	0.004	0.004	0.005	0.005	0.005	0.006	0.005

We show the standard errors of RF rates of QFM and QMC over the 20 replicates of data under various model conditions. We varied the number of taxa (n), the number of quartets (q), and the percentage of consistent quartets (c).

Table S2. Standard error of QFM and QMC under the noise-free model conditions.

n	q	Standard error	
		c = 100%	
		QFM	QMC
25	125	0.027	0.022
25	625	0.007	0.008
25	8208	0	0
50	354	0.021	0.017
50	2500	0.007	0.010
50	57164	0	0
100	1000	0.013	0.007
100	10000	0.011	0.010
100	398108	0	0
200	2829	0.006	0.004
200	40000	0.008	0.009
300	5197	0.005	0.003
300	90000	0.007	0.007
400	8000	0.004	0.002
400	160000	0.006	0.005
500	11181	0.003	0.002
500	250000	0.003	0.004

Standard error of RF rates of QFM and QMC over the 20 replicates of data under the noise-free model conditions (c = 100%). We varied the number of taxa (n) and the number of quartets (q).

Table S3. Statistical significance of the differences between QFM and QMC.

n	q	p-values				
		c = 70%	c = 80%	c = 90%	c = 95%	c = 100%
25	125	0.389	0.139	0.426	0.288	0.300
25	625	0.050	0.052	**0.013**	0.090	0.156
25	8208	0.500	0.500	0.500	0.500	0.500
50	354	0.170	0.175	0.470	0.301	0.354
50	2500	**0.022**	**0.0005**	0.185	0.058	0.441
50	57164	0.250	0.500	0.500	0.500	0.500
100	1000	0.060	0.068	0.426	**0.024**	0.068
100	10000	**0.001**	**0.015**	0.063	**0.040**	0.239
100	398108	0.383	0.500	0.500	0.500	0.500
200	2829	0.094	0.078	**0.001**	**0.007**	**0.0002**
200	40000	**0.018**	**0.015**	**0.003**	**0.011**	0.500
300	5197	0.222	**0.010**	**0.00007**	**0.00008**	**0.001**
300	90000	**0.003**	**0.006**	**0.002**	0.109	0.148
400	8000	**0.036**	**0.00004**	**0.00005**	**0.00007**	**0.00005**
400	160000	**0.001**	**0.0002**	**0.020**	0.084	0.470
500	11181	0.210	**0.001**	**0.00006**	**0.00007**	**0.00004**
500	250000	**0.0003**	**0.0003**	**0.002**	**0.0004**	**0.001**

We calculated the p-values using the Wilcoxon signed-rank test (with $\alpha = 0.05$) for all the model conditions. Here n is the number of taxa, q is the number of quartets and c is the percentage of consistent quartets. The p-values, which indicate the statistically significant differences (i.e., $p < 0.05$), are shown in bold face. The differences are statistically significant in 38 cases (in most of these cases, $p << 0.05$), and QFM is found to be better than QMC on all of these 38 model conditions. The differences between QFM and QMC on the 7 cases, where QMC was found to be better than QFM, are not statistically significant.

Table S4. Algorithm MFM(P, Q)

```
(P_a0, P_b0) ← INITIAL_PARTITION(P, Q)
repeat always
   FREE_LOCKS(P)     //set the status of each taxon free
   CLEAR_LOG()     //maintain a log file, initially blank
   i ← 1
  while there is a free taxon do
    begin
        find a free taxon t_i so that Gain(t_i, (P_a(i-1), P_b(i-1))) is maximum
        break tie in case of multiple candidates
        transfer t_i to the other partition
        update (P_a(i-1), P_b(i-1)) to (P_a_i, P_b_i)
        LOCK (t_i)     //set the status of taxon t_i locked
        LOG_RECORD (t_i, Gain(t_i, (P_a(i-1), P_b(i-1))))   //write on log file
        increment i
     end do
  check the log file and find MCGain(t_1, t_2, ..., t_n) and t_m     //cumulative gain is maximum at the m-th transfer
  if MCGain(t_1, t_2, ..., t_n) > 0
    begin
        set new (P_a0, P_b0) by rolling back the transfers that occurred
        after the transfer of t_m
        continue with the the loop
  end if
  else
    begin
        terminate the algorithm and output current partition
  end else
end repeat
```

Modified FM (MFM) Bipartition Algorithm.

Time Complexity of MFM Bipartition Algorithm

QFM is a divide and conquer approach. The time required at a conquer step is negligible compared to the time required at a divide step. The key contributing factor in the time required by a divide step is the time taken to make a bipartition of the set of taxa. Now we derive the theoretical running time of our bipartition algorithm MFM (P, Q), where P is a set of taxa and Q is a set of quartets over P. Let, n and m be the cardinality of taxa set P and the quartet set Q respectively. We first derive the running time for the *Initial Partition.*

Initial Partition: First, we count the frequency of the distinct quartets in Q and sort Q by frequency count. The counting and the sorting step requires $O(m^2)$ running time. Then, we check each quartet $q \in Q$ and insert each of its 4 taxa either in P_a or in P_b by checking the existing elements of P_a and P_b. The length of P_a or P_b is bounded by $O(n)$, so the time required to insert taxa of each quartet is $O(n)$. For m quartets, the required time is $O(nm)$. Overall, the total time complexity of initial bipartition is $O(m^2) + O(nm)$.

Next, we explain the time required for the remaining part of MFM, which is accomplished in several iterations. Let, the maximum cumulative gain becomes less or equal to 0 in k iterations. The time complexity per iteration is described below.

- Gain Measure of a Partition: The gain of a new partition is the difference between its score and the score of initial partition. The difference is measured in $O(1)$ time. We need to find out the time required to calculate the partition score of a partition (P_a, P_b). To calculate score, each $q \in Q$ is checked against the partition (P_a, P_b), which takes $O(n)$ time since the length of P_a or P_b is bounded by $O(n)$. Hence to check m quartets, hence to calculate partition score, $O(nm)$ time is required.

- SELECT_FREE_TAXON(P): One taxon is selected among the free taxa. For each free taxon *Gain* is measured and the taxa with maximum gain is selected. There are n free taxa initially, so this step requires $n \times O(nm) = O(n^2m)$ time. The selected taxon is made *locked.*

- There are n free taxon initially. Each taxon is selected and locked one after another. So the total time complexity to lock all the taxa $= n \times O(n^2m) = O(n^3m)$.

- Each locked taxon has a gain associated with it. When all taxa are locked, cumulative gain and maximum cumulative gain are calculated. These operations take $O(n)$ time.

Overall the running time for one iteration is $O(n^3m) + O(n) = O(n^3m)$. For k iterations, the time complexity becomes $O(n^3mk)$. Taking the time required by the initial partition into account, a divide step requires $O(n^3mk) + O(m^2)$ time.
